# Antimicrobial peptide LL-37 is bactericidal against *Staphylococcus aureus* biofilms

**DOI:** 10.1371/journal.pone.0216676

**Published:** 2019-06-06

**Authors:** Jason Kang, Matthew J. Dietz, Bingyun Li

**Affiliations:** Department of Orthopaedics, School of Medicine, West Virginia University, Morgantown, WV, United States of America; Brandeis University, UNITED STATES

## Abstract

Our current challenge in the management of prosthetic joint infection is the eradication of biofilms which has driven the need for improved antimicrobial agents and regimens. In this study, the antimicrobial peptide, LL-37, and silver nanoparticles (AgNPs) were investigated for their antimicrobial efficacies against *Staphylococcus aureus* (*S*. *aureus*), a microorganism commonly implicated in biofilm-related infections. These antimicrobials were compared to conventional antibiotics and combination treatments with rifampin. Using a Centers for Disease Control reactor, 24 h *S*. *aureus* biofilms were formed on cobalt-chromium discs and the anti-biofilm activity was determined by quantifying the amount of colony forming units following treatments. We found that LL-37 was the most efficacious antimicrobial agent with a more than 4 log reduction in colony counts. In comparison, silver nanoparticles and conventional antibiotics were not as efficacious, with a less than 1 log reduction in colony counts. Antimicrobial combination treatments with rifampin significantly increased the log reduction for AgNPs and gentamicin, although still significantly less than LL-37 in isolation. Furthermore, kinetic studies revealed the rapid elimination of *S*. *aureus* biofilm with LL-37. Collectively, the results of this study demonstrated that LL-37 was an effective agent against *S*. *aureus* biofilms and may have potential clinical applications in the eradication of biofilms and treatment of prosthetic joint infection.

## Introduction

Primary arthroplasty, such as total knee arthroplasty (TKA), is one of the most commonly performed orthopedic procedures. The number of TKAs performed in the US is expected to increase by 143% from 2012 to 2050 with 1.5 million cases per year by 2050 [1]. This procedure provides symptomatic pain relief and helps improve mobility. However, a small proportion of these patients will become infected, as between 0.5 to 2% of these procedures may result in prosthetic joint infection (PJI), an infection of the prosthesis, joint, and adjacent tissue [2–6]. This type of implant failure causes considerable morbidity and is associated with significant financial costs to the healthcare system. In 2009, the total estimated cost for treating PJI was about $566 million [2]. Thus, with an increasing number of primary arthroplasties being performed each year, we can expect the incidence of PJI and its associated burdens to rise as well.

*Staphylococcus aureus* (*S*. *aureus*) is the most frequently isolated microorganism in PJI [7]. The persistence of *S*. *aureus* in PJI is attributed to many factors, which include increasing antibiotic resistance [8], intracellular survival of bacteria [9–11], and formation of biofilms [12]. In particular, biofilms are believed to have a large role in the pathogenesis of PJI, as foreign medical devices, such as prostheses, are prone to biofilm formation, and biofilms have many properties that make treatment difficult, especially with antibiotics [13].

The goals of PJI treatment are aimed at eradicating the infection. This is best accomplished through a combination of surgical and antimicrobial therapies. Depending on the severity and timing of the infection, surgical options may include resection arthroplasty with re-implantation, in a one- or two-stage exchange, or debridement with retention of prosthesis. These surgical procedures are typically accompanied by four to six weeks of parenteral antibiotics, followed by three to six months of oral antibiotic therapy in some cases [14]. However, there are many challenges in treating these infections with antibiotics. Long-term, systemic administration of antibiotics may cause adverse effects [15]. Furthermore, biofilms have many properties that may limit the efficacy of antibiotics and can generate resistance, allowing infection to recur [13]. As a result, current regimens of treatment have relatively high failure rates. Overall re-infection rates after first-line treatment [1-stage, 2-stage, or irrigation and debridement (I&D)] are 26% after one year and 35.8% after six years [16]. The five-year infection free survival rate following I&D with oral antibiotic therapy has been reported as 68.5% [17]. Thus, it is important to continue to identify antimicrobial agents that could be more efficacious (compared to conventional antibiotics) in treating infections thereby reducing the incidence of re-infection.

One promising antimicrobial agent is the antimicrobial peptide (AMP) LL-37. Rapid and efficient methods have been developed over the years to yield recombinant forms of LL-37, allowing for increased clinical and functional characterization, on what is an otherwise prohibitively expensive peptide [18,19]. Previous studies in the literature regarding the efficacy of LL-37 against pre-formed biofilms vary. Some studies suggest that LL-37 does not disrupt pre-formed biofilms, inhibit bacterial attachment, or prevent early biofilm formation [20,21]. In contrast, other studies have demonstrated that LL-37 can disrupt 24 and 48 h mature *S*. *aureus* biofilms [22]. In our evaluation, biofilms are formed using the Centers for Disease Control (CDC) biofilm reactor (CBR), which better replicates biofilms that are found clinically. There remains a need to further elucidate the antimicrobial properties of LL-37 and its potential use in combination with conventional antibiotics against CBR-formed biofilms.

The objectives of this study were to determine the *in vitro* treatment of *S*. *aureus* biofilms with two non-antibiotic antimicrobial agents (i.e. LL-37 and silver nanoparticles (AgNPs)) and to compare them with conventional antibiotics (e.g. gentamicin, vancomycin, and rifampin). *S*. *aureus* biofilms were grown on cobalt chrome (Co-Cr) discs and the efficacy of the treatments of various antimicrobial agents were evaluated by quantifying the number of viable bacteria. We hypothesized that LL-37 and AgNPs would be more efficacious than conventional antibiotics (i.e. gentamicin, vancomycin, and rifampin) in eradicating *S*. *aureus* biofilms.

## Materials and methods

### Bacterial strain

A clinical isolate of *S*. *aureus* (SA 1004) was previously obtained from a patient’s chronic wound from Ruby Memorial Hospital in Morgantown, WV [23] and was used in this study. Susceptibility tests revealed that the isolate was resistant to ampicillin, cefoxitin, and penicillin, and susceptible to gentamicin, vancomycin, rifampin, cefazolin, clindamycin, ciprofloxacin, levofloxacin, erythromycin, linezolid, oxacillin, moxifloxacin, tigecycline, and tetracycline.

### Minimum inhibitory concentration

The minimum inhibitory concentration (MIC) of human LL-37 (Sigma Aldrich, St. Louis, MO), AgNPs (40 nm, polyvinylpyrrolidone-capped) (nanoComposix, Inc., San Diego, CA), and conventional antibiotics (i.e. gentamicin, vancomycin, rifampin, clindamycin, cefazolin) (Sigma Aldrich) were conducted by the broth microdilution technique based on the guidelines set by the Clinical and Laboratory Standards Institute (CLSI) [24]. Briefly, in 96-well microtiter plates, increasing concentrations of antimicrobial agents were added to each well, starting at 0.25 μM, and doubling in concentration until reaching a final concentration of 128 μM. Equal volumes of bacterial inoculum grown in Muller-Hinton Broth (MHB) (Becton Dickinson, Sparks, MD) containing 1×10^6^ colony forming units (CFUs)/ml were subsequently added. The 96-well plate was incubated at 37°C for 18 h. After incubation, the MIC was read as the lowest concentration of antimicrobial agent that visibly inhibited bacterial growth.

### Biofilm formation

To prepare an inoculum, colonies of *S*. *aureus* were suspended in brain heart infusion (BHI) (Becton Dickinson) and incubated for 18 h at 37°C. The inoculum was diluted to a 0.5 McFarland standard with fresh BHI and seeded in a CBR (BioSurfaces Technologies, Bozeman, MT). Biofilms were formed on surgical grade Co-Cr discs (BioSurfaces Technologies) with a total surface area of 1.57 cm^2^. The CBR was run for 24 h at 37°C with continuous stirring at 120 rpm.

### Dose-response and kinetics of antimicrobial agents

Following 24 h biofilm formation, Co-Cr discs were removed from the CBR, placed in round bottom polystyrene tubes, and washed in phosphate buffered saline (PBS) (Corning, Manassas, VA) to remove planktonic bacteria. The dose-response and kinetics of LL-37, AgNPs, conventional antibiotics (gentamicin, vancomycin, and rifampin) and combinations with rifampin were investigated. For the dose response studies, LL-37 (1.75, 2.5, 3.75, 5, 10, and 100 μM), AgNPs (100, 500, and 1000 μM), gentamicin (100, 500, and 1000 μM), vancomycin (100, 500, and 1000 μM), rifampin (100, 500, and 1000 μM), AgNPs + rifampin (100 μM each), gentamicin + rifampin (100 μM each), vancomycin + rifampin (100 μM each), and controls (plain PBS) were applied and incubated at 37°C for 60 min. For the kinetic studies, LL-37 (10 μM), rifampin (100 μM), gentamicin (100 μM), gentamicin + rifampin (100 μM each), and controls (plain PBS) were applied at incubation intervals of 5, 30, and 60 min at 37°C. Shorter incubation intervals of 5 and 30 minutes were chosen, as our previous study indicated that LL-37 was fast acting at these time points [23]. For all assays, the total treatment volume was 1 mL. Following treatment, the discs were washed in PBS to remove planktonic bacteria and residual chemicals from the treatment, and were transferred to another tube containing 2 mL of BHI. The discs were then sonicated for 10 min at 50 Hz in a TRU-SWEEP Ultrasonic Cleaner (CREST Ultrasonics, Trenton, NJ) and vortexed for 2 min to break up the remaining biofilm. The samples were serially diluted in PBS to 10^−1^, 10^−2^, and 10^−3^ and plated on 5% sheep blood agar plates (Remel, Lenexa, KS). The plates were incubated for 24 h at 37°C and the number of CFUs were quantified. All samples were performed in triplicate and the average was reported. The outcomes were reported as log reduction, which was calculated by subtracting the difference between the log CFU/cm^2^ of the control and the log CFU/cm^2^ of the treated samples. Bactericidal activity was defined as a more than 3 log reduction in colony count from the initial inoculum [25].

### Statistical analysis

All data were presented as means ± standard deviations. Differences in log reduction of all treatments were assessed using JMP-V14 statistical software (SAS Institute, Cary, NC). Comparisons between groups were conducted with one-way ANOVA followed by Tukey’s honestly significant difference (HSD) test. Combination therapy analysis was performed by taking the logarithm of the percent colonies remaining after treatment and comparing the combined and individual treatments and their interactions for significance with one-way ANOVA. A p-value < 0.05 was considered statistically significant.

## Results

### Minimum inhibitory concentration

The antimicrobial susceptibility of LL-37, AgNPs, gentamicin, vancomycin, rifampin, clindamycin, and cefazolin was determined against a clinical strain of *S*. *aureus*. The MIC of LL-37 was determined to be 32 μM (Table 1). In comparison, the MIC of AgNPs, gentamicin, vancomycin, rifampin, clindamycin, and cefazolin was found to be >128, 0.5, 0.5, <0.25, 0.5, and 2 μM, respectively (Table 1).

**Table 1:** Minimum inhibitory concentration (MIC) of novel and conventional antimicrobial agents against planktonic *S*. *aureus*.

Treatment	MIC (μM)
**LL-37**	32
**AgNPs**	128
**Gentamicin**	0.5
**Vancomycin**	0.5
**Rifampin**	0.25
**Clindamycin**	0.5
**Cefazolin**	2

### Dose-response effect on biofilms

**Fig 1:**
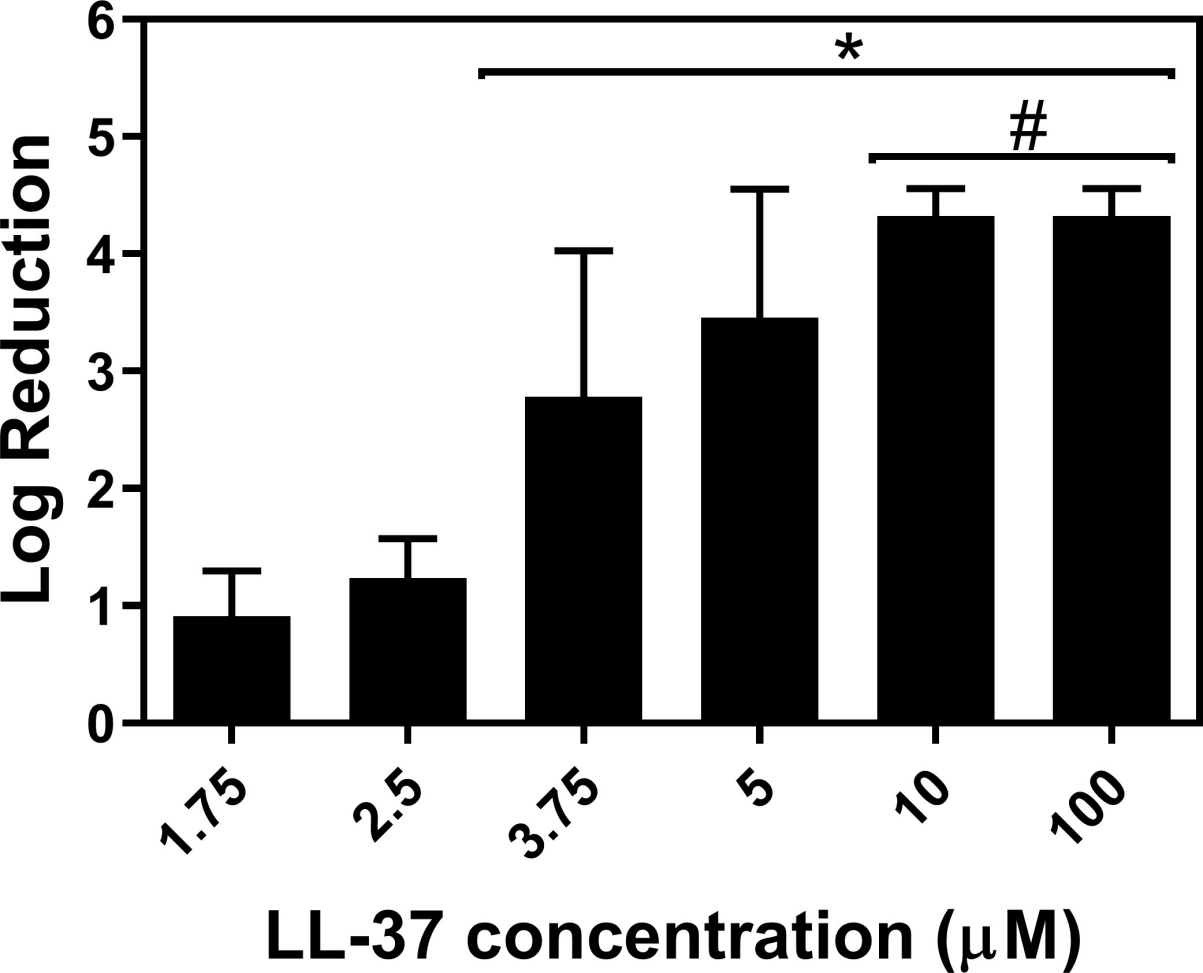
Dose-response effect of LL-37 against *S*. *aureus* biofilms. The antimicrobial peptide, LL-37, was used to treat 24 h *S*. *aureus* biofilms for 60 min. LL-37 was found to present high antimicrobial activity against *S*. *aureus* biofilms, with a more than 4 log reduction at concentrations starting at 10 μM. * Statistically significant from 1.75 and 2.5 μM; # statistically significant from 3.75 and 5 μM.

**Fig 2:**
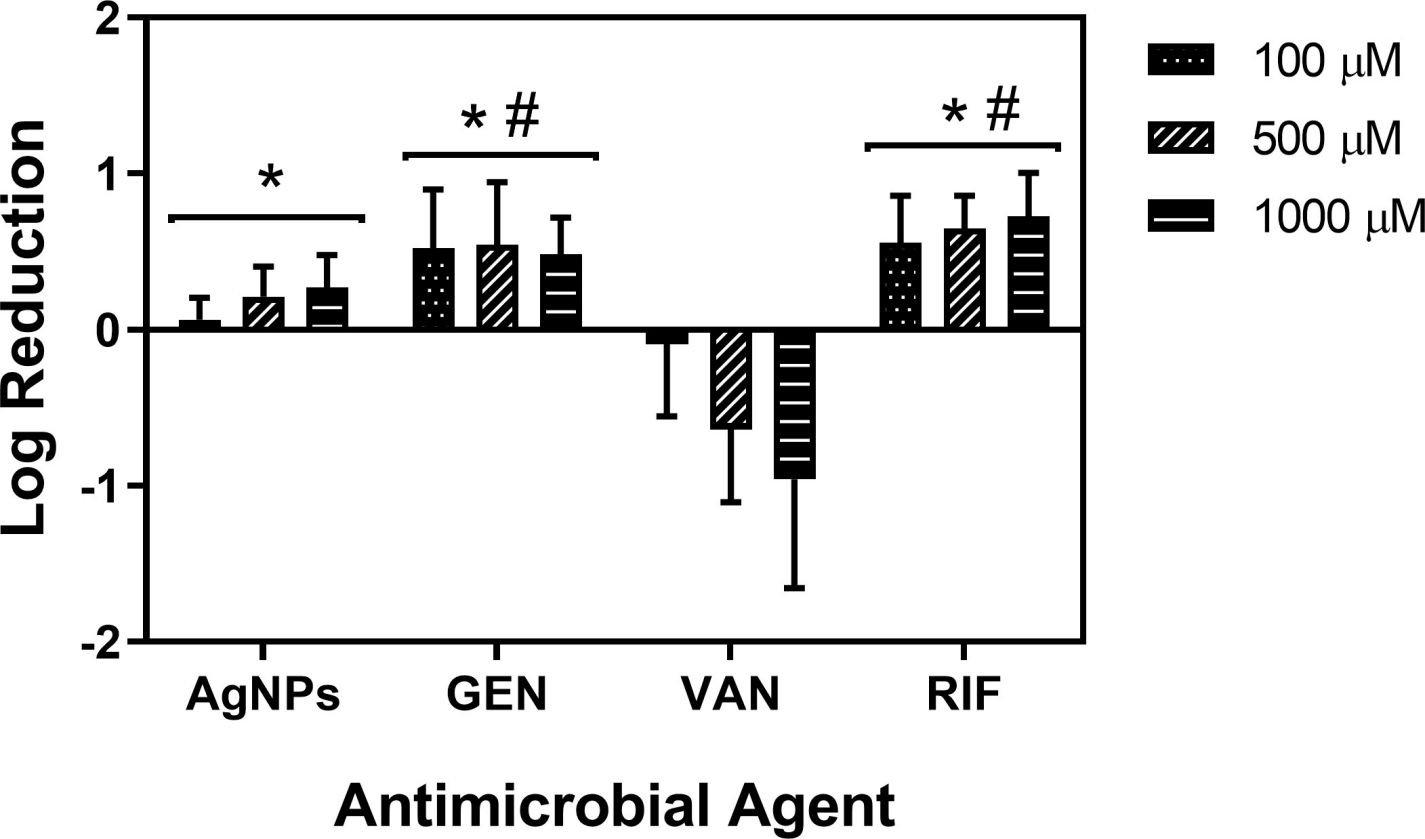
Dose-response effect of AgNPs and conventional antibiotics against *S*. *aureus* biofilms. Conventional antibiotics (gentamicin, vancomycin, and rifampin) and AgNPs were used to treat 24 h *S*. *aureus* biofilms for 60 min. At concentrations up to 1000 μM, all treatments were found to present a less than 1 log reduction. * Statistically significant from VAN; # statistically significant from AgNPs. Abbreviations GEN, gentamicin; VAN, vancomycin; RIF, rifampin.

**Fig 3:**
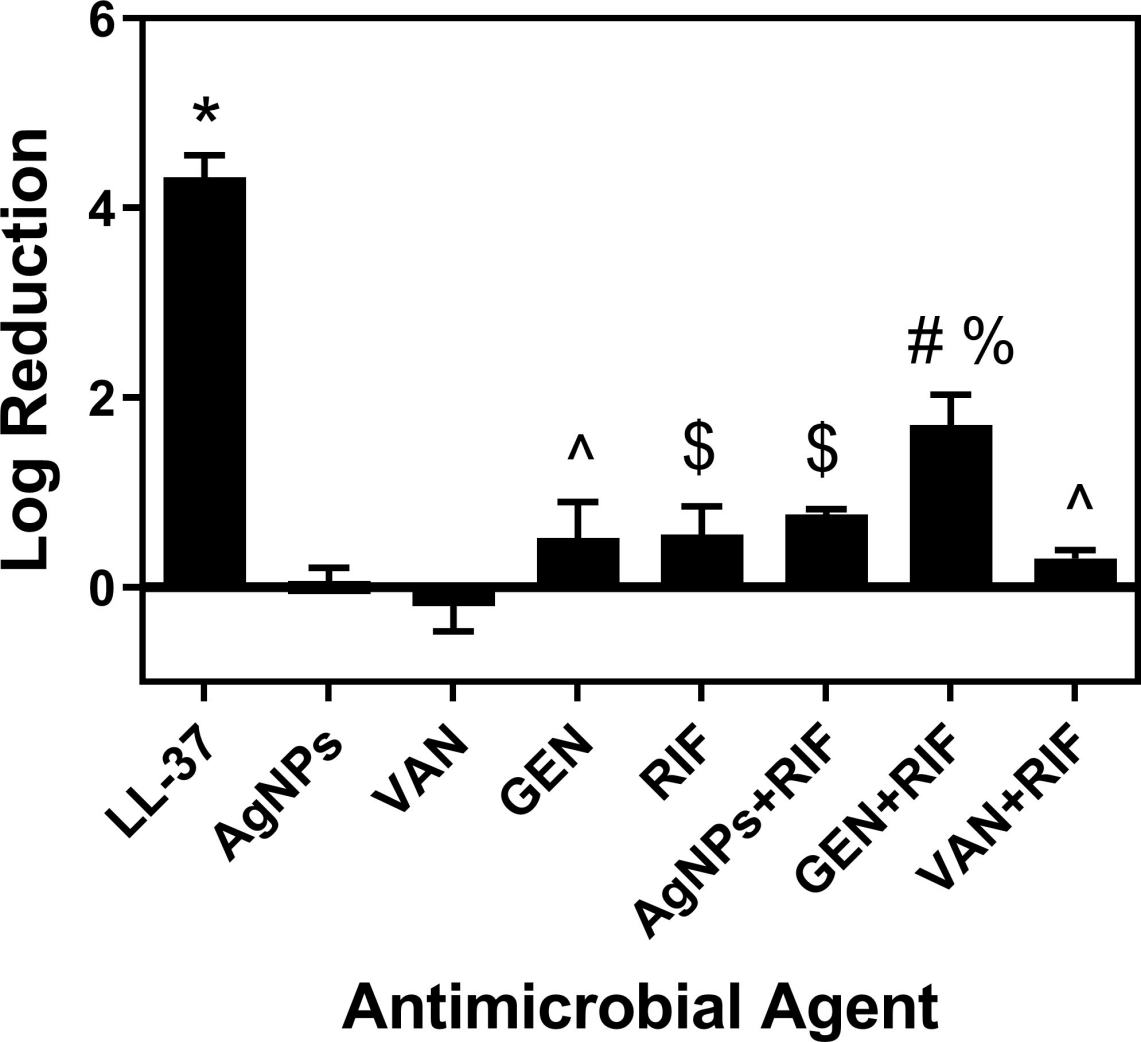
Combination treatment of antimicrobial agents with rifampin against *S*. *aureus* biofilms and comparison to LL-37 at 100 μM. Combination treatments with rifampin at concentrations of 100 μM were used to treat 24 h *S*. *aureus* biofilms for 60 min. The combination of GEN+RIF was the most significant combinatory treatment investigated, with a log reduction of 1.71. In addition, all antimicrobial agents investigated in this study were displayed at a concentration of 100 μM, where it was observed that LL-37 had the greatest log reduction against *S*. *aureus* biofilms. * Statistically significant from AgNPs, VAN, GEN, RIF, AgNPs+RIF, GEN+RIF, VAN+RIF; # statistically significant from AgNPs, VAN, GEN, RIF, AgNPs+RIF, VAN+RIF; $ statistically significant from AgNPs, VAN; ^ statistically significant from VAN; % statistically significant from the combination of GEN and RIF.

Treatment of 24 h *S*. *aureus* biofilms formed on Co-Cr discs with LL-37, AgNPs, conventional antibiotics, and combination treatments with rifampin were investigated. Treatment with LL-37 at concentrations of 1.75, 2.5, 3.75, 5, 10, and 100 μM resulted in a log reduction of 0.9, 1.2, 2.8, 3.5, 4.3, and 4.3, respectively (Fig 1). In comparison, there was a low eradication of *S*. *aureus* biofilm for AgNPs, conventional antibiotics, and combinations with rifampin. At concentrations as high as 1000 μM, there was a less than 1 log reduction in CFU for AgNPs, gentamicin, vancomycin, and rifampin (Fig 2). Combination treatments of AgNPs + rifampin, gentamicin + rifampin, and vancomycin + rifampin resulted in log reductions of 0.76, 1.71, and 0.30, respectively (Fig 3). The combination of gentamicin + rifampin demonstrated a significant increase in log reduction over gentamicin and rifampin in isolation. Though gentamicin + rifampin was found to be the only combinatory treatment with statistical significance over its combined individual counterparts, its log reduction was still significantly less than that of LL-37 in isolation (Fig 3).

### Kinetics analysis

**Fig 4:**
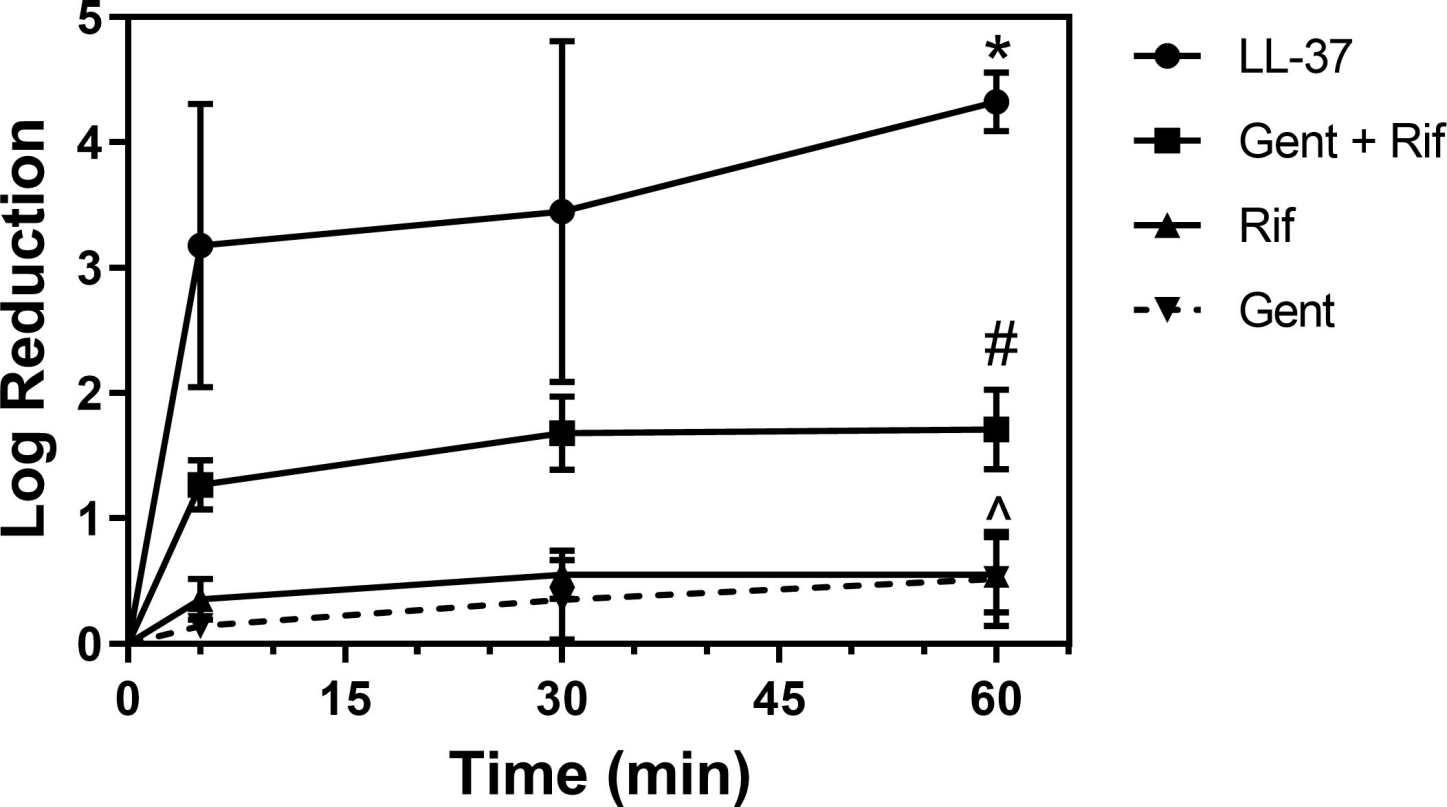
Kinetics of LL-37 against *S*. *aureus* biofilms with comparison to conventional antibiotics. Kinetic studies were performed at 5, 30, and 60 min against 24 h *S*. *aureus* biofilms with the antimicrobial agents LL-37 (10 μM), GEN (100 μM), RIF (100 μM), and GEN+RIF (100 μM). LL-37 was found to be fast-acting against *S*. *aureus* biofilms with a more than 3 log reduction within 5 min of treatment time. * LL-37 at 60 min statistically significant from LL-37 at 5 min; # GEN+RIF at 60 min statistically significant from GEN+RIF at 5 min; ^ GEN at 60 min statistically significant from GEN at 5 min.

The log reduction kinetics of LL-37 was compared to gentamicin, rifampin, and gentamicin + rifampin. LL-37 had a 3.2 log reduction in CFU within 5 min of treatment time (Fig 4). In contrast, the log reduction at 5 min for gentamicin, rifampin, and gentamicin + rifampin was 0.14, 0.36, and 1.3, respectively (Fig 4). Increasing the treatment time up to 60 min for LL-37, gentamicin + rifampin, and gentamicin demonstrated a significant increase in log reductions and demonstrated the presence of time trend for these antimicrobial agents (Fig 4).

## Discussion

There are hundreds of antimicrobial peptides (AMPs) in humans that are part of the innate immune system [26]. Of interest in this study is LL-37, a cathelicidin-derived AMP, which functions as an immunomodulatory agent and has direct antimicrobial activities against many Gram-positive and -negative bacteria, including *S*. *aureus* [27]. In this study, we investigated the antimicrobial efficacy of LL-37 against *S*. *aureus* biofilms. The biofilm was created in the CBR, which is a rugged system that allows for similar replication of biofilms found *in vivo*, with fluid sheer dynamics in the growth medium [28]. In addition, more practical applications may be achieved with this method, as biofilms can be formed on metal substrates, such as Co-Cr, which is a common orthopedic material used in prosthetic implants.

This study demonstrated that LL-37 has excellent antimicrobial activity against 24 h *S*. *aureus* biofilms. Our results showed that, at sub-MIC concentrations, starting at 10 μM, LL-37 was bactericidal against *S*. *aureus* biofilms with a more than 4 log reduction in colony counts. No change in log reduction was observed up to 100 μM. In addition, LL-37 was also fast acting against *S*. *aureus* biofilms, requiring low incubation times (e.g. 5 min) to achieve a more than 3 log reduction in colony counts. Though the mechanism that LL-37 employs against biofilms is still largely unknown, it is likely that LL-37 penetrates the biofilm and has antimicrobial activity against the embedded bacteria [29]. Other mechanisms may include the decreased attachment of planktonic bacteria to prevent biofilm formation and down-regulation of quorum-sensing systems related to biofilm development and maintenance [30].

This study also investigated the effect of AgNPs, conventional antibiotics, and their combinations against *S*. *aureus* biofilms. Gentamicin, vancomycin, and rifampin were chosen as conventional antibiotics to study due to their widespread use in the treatment of orthopedic-related infections. Vancomycin is commonly chosen among physicians as a first-line empiric antibiotic due to its excellent Gram-positive coverage and action against methicillin-resistant *S*. *aureus* (MRSA) [14,31]. Gentamicin is also a common empiric agent due to its broad spectrum of action, particularly against Gram-negative suspected infections, and can also be found as an additive in cement spacers and polymethylmethacrylate (PMMA) beads [32,33]. Rifampin is a frequently recommended combinatorial antibiotic in PJI due to its ability to penetrate biofilms and activity against biofilm microorganisms [14,34]. Finally, as a novel antimicrobial agent, AgNPs are gaining popularity due to their broad spectrum of action and anti-biofilm efficacy [35,36], and are even being used in orthopedic applications such as coatings for prosthetic implants and external fixation pins [37].

In our study, LL-37 was found to have much greater anti-biofilm activity in comparison to AgNPs and conventional antibiotics. At concentrations of 1000 μM, which is 2000 times to 4000 times the MIC of conventional antibiotics, there was a less than 1 log reduction in colony counts after treatment with AgNPs, gentamicin, vancomycin, and rifampin. Combination treatments with rifampin demonstrated a significant increase in log reduction for AgNPs and gentamicin. Interestingly, there was a negative log reduction observed with vancomycin. The limited response to vancomycin is concerning, as it is frequently used in the management of infections suspected to be caused by MRSA. A few other studies have reported an increase in biofilm density after vancomycin treatment, which may be due to non-responsive bacterial strains to vancomycin [38] or sub-inhibitory concentrations during treatment [39]. Vancomycin has been shown to have a time-dependent nature against *S*. *aureus* biofilms [40], as the relatively brief treatment duration in this study may not be adequate and could be the reason for the negative log reduction.

Our findings demonstrate some of the challenges in eradicating biofilms with AgNPs and conventional antibiotics. Although AgNPs and antibiotics are frequently being used in the treatment and prevention of PJI, our study suggests that these antimicrobials result in incomplete eradication of the *S*. *aureus* biofilm, which may ultimately lead to chronic and recurrent infection. In comparison, LL-37 was shown to be fast-acting and have a superior eradication of biofilms, with a greater log reduction in colony counts at sub-MIC concentrations. The higher efficacy against biofilms of LL-37 was consistent with the findings that some antimicrobial peptides are more effective against biofilms compared to other antimicrobial agents including conventional antibiotics, and LL-37 was found to be more potent against intracellular *S*. *aureus*, similar to biofilm persister cells, compared to conventional antibiotics [23]. As a result, LL-37 may be a better choice as a therapeutic agent against biofilm-related infections and may find increased applications toward infection treatment and prevention.

Some concerns with the use of AMPs as therapeutic agents include its inactivation in environments of elevated salt concentration, which may limit its use *in vivo*, as studies have demonstrated reduced activity of LL-37 under physiological and high salt conditions [41,42]. Our study demonstrated that LL-37 had a MIC of 32 μM, whose relative increase in comparison to antibiotics could be attributed to inactivation under media conditions. Comparable studies report a similar MIC for LL-37. Depending on the strain of bacteria, reported MICs for LL-37 have ranged from 6.25 to >128 μM [21,22]. Although LL-37 may lose its antimicrobial activity under elevated salt conditions, it may still retain its anti-biofilm properties. Dean *et al*. reported that, under conditions of high salt, LL-37 has significant inhibition of *S*. *aureus* biofilm at sub-antimicrobial concentrations [43]. Furthermore, Chennupati *et al*. demonstrated the eradication of *Pseudomonas aeruginosa* (*P*. *aeruginosa*) biofilm with LL-37 in an animal model of sinusitis [44].

Another concern with the use of AMPs is its potential for bacterial resistance. Some studies have reported that the use of LL-37 leads to a selection of AMP-resistant pathogens. Leszczyńska *et al*. reported that clinical *S*. *aureus* strains developed increased resistance after three passages with sub-MICs of LL-37 [45]. Lofton *et al*. found that AMP-resistant strains of *Salmonella typhimurium* were generated after prolonged exposure to LL-37, with an increase in MIC after 490–553 generations [46]. Furthermore, Strempel *et al*. showed that physiologic concentrations of LL-37 upregulated resistance factors in *P*. *aeruginosa*, such as increased production of quorum-sensing molecules, secreted virulence factors, lipopolysaccharide modification, and genes encoding multidrug efflux pumps [47]. In general, mechanisms of resistance may include membrane modifications to reduce the binding of AMPs, efflux pumps, and proteolytic degradation [48]. Though the generation of AMP-resistant bacteria is concerning and warrants continued evaluation, AMPs are less susceptible to bacterial resistance when compared to antibiotics, due to several mechanisms of inhibiting bacteria including membrane disruption and inhibition of cellular processes [49].

One limitation of this study is the need to determine the potential cytotoxicity of LL-37 against eukaryotic cell lines. Previous studies have investigated this issue, and Johansson *et al*. reported that LL-37 displayed toxicity toward T-lymphocyte cell lines at concentrations of 13 to 25 μM [50]. Furthermore, Säll *et al*. reported that LL-37 reduced the number and viability of human MG63 osteoblasts at an IC_50_ value of about 5 μM [51]. There are many strategies that exist in reducing its cytotoxicity. It is possible to reduce the cytotoxicity of LL-37 through truncation of its N-terminal amino acid residues [52]. In addition, LL-37 selectively permeabilizes apoptotic cells over viable cells [53]. There are also eukaryotic host cell defenses, such as the globular C1q receptor, p33, which antagonizes and binds to LL-37 [54]. The determination of cytotoxicity is important in delineating potential therapeutic windows. Considering previous cytotoxic reports, a narrow window may exist for LL-37 in the low micromolar range and is especially likely with the body of literature detailing its reduction in cytotoxicity.

In summary, our study demonstrates the excellent antimicrobial activity and kinetics of LL-37 against *S*. *aureus* biofilms. In comparison, conventional antibiotics and AgNPs were not as efficacious in eradicating *S*. *aureus* biofilms. Though the log reduction with the addition of rifampin was significantly increased for AgNPs and gentamicin, it was still significantly less than LL-37. The treatment model utilized in this study allows for insight toward the treatment of clinical applications, such as the treatment of PJI. This study identified current challenges in treating PJI with conventional antibiotics and suggests that LL-37 may be an alternative therapeutic agent in eradicating infection, particularly those related to biofilm formation.

## Supporting information

S1 Datasets Data sets for Table 1 and Figs 1–4 are available. (DOCX)
