# Supplementary material for: Antimicrobial peptide LL-37 is bactericidal against Staphylococcus aureus biofilms
Source: PLoS One. 2019 Jun 6;14(6):e0216676. doi: 10.1371/journal.pone.0216676 (PMC6553709; doi:10.1371/journal.pone.0216676)
Supplement: S1 Datasets — (DOCX) [file pone.0216676.s001.docx]

Supporting Information:

**Antimicrobial peptide LL-37 is bactericidal against *Staphylococcus aureus* biofilms**

Jason Kang^1^, Matthew J. Dietz^1*^, Bingyun Li^1*^

^1^Department of Orthopaedics, School of Medicine, West Virginia University, Morgantown, WV 26506, USA

Data sets:

Table 1:

Fig. 1:

Fig. 2:

Fig. 3:

Fig. 4:
